# Understanding the impact of prior depressive and anxiety symptoms and autism diagnosis on menopause symptoms

**DOI:** 10.1177/17455057261446945

**Published:** 2026-05-07

**Authors:** Rebecca A. Charlton, Nell Fahey, William Mandy, Francesca Happé, Gavin R. Stewart

**Affiliations:** 1 Psychology, School of Mind, Body & Society, 4898 Goldsmiths University of London, London, UK; 2 Social, Genetic and Developmental Psychiatry Centre, Psychology & Neuroscience, Institute of Psychiatry, 47957 King’s College London, London, UK; 3 Department of Clinical, Educational and Health Psychology, 224223 UCL, London, UK

**Keywords:** autism, menopause, depression, anxiety, longitudinal

## Abstract

**Background:**

There is growing awareness from qualitative research studies that menopause may be particularly challenging for autistic people. Research in the general population suggests that preexisting conditions may be a risk factor for negative experiences during menopause. However, there are limited studies examining variables associated with experiences of menopause symptoms for autistic people.

**Objectives:**

To explore whether pre-existing depression and anxiety symptoms impact menopause symptoms for autistic and non-autistic people.

**Design:**

The study design is classified as STROBE.

**Methods:**

Autistic (n=52) and non-autistic (n=28) people assigned female at birth participated in the longitudinal AgeWellAutism (AWA) study. They reported self-report depression and anxiety symptoms at baseline and menopause symptoms (Greene Climacteric Scale) at follow-up after 4.5 years. Age, autism diagnosis and autism characteristics of mentalising difficulties, sensory reactivity, and social anxiety (subscales from the self-reported Ritvo Autism and Asperger Diagnostic Scale, RAADS) were recorded.

**Results:**

Regression analyses were conducted with menopause symptoms as the dependent variable and as independent variables: Step 1, age; Step 2, baseline depression and anxiety symptoms, Step 3, autism diagnosis (alternative step 3, RAADS subscales). In the final regression model, younger age, higher baseline depression and autism diagnosis contributed significantly to explaining menopause symptom severity. In the alternative model (including subscales of the RAADS), younger age, higher baseline depression, mentalising difficulties and sensory reactivity contributed significantly to explaining menopause symptom severity.

**Conclusions:**

Results align with research from the general population suggesting that a history of depression symptoms increases risk of negative experiences during menopause, furthermore, being autistic confers additional risk. Further studies examining the influence of lifetime experiences on menopause symptoms for neurodivergent people are required to better understand and mitigate risk during this critical time.

## Introduction

Recognition of autism in women and girls has increased in recent years.^1,2^ There is a growing awareness from qualitative research that menopause may be especially challenging for autistic people. Autistic people often describe increased challenges relating to psychological symptoms and mental health during menopause, with autism frequently described as a factor that interacts with menopause symptoms to cause additional difficulties.^3–5^ Importantly, menopause has been described as heightening existing sensory, cognitive and social challenges.^4^ For some, menopause may be the impetus to seek an autism diagnosis although this is likely to interact with the challenges (and duration) of the diagnostic process.^6,7^ Despite menopause being described as a clearly challenging time, autistic people also describe difficulties accessing appropriate information and support (see academic papers,^3,5,8^ and a UK government report,^9^). A survey of (n=136) autistic people demonstrated high levels of discomfort discussing reproductive and sexual health issues with healthcare professionals, and reported lack of awareness and accommodation for autistic differences.^8^ In combination with many autistic people’s lifelong difficulties accessing and trusting healthcare professionals and healthcare professionals’ own lack of confidence working with autistic people, sensitivities around discussing reproductive health needs may create a barrier to accessing much needed support.^4,10^ In previous work comparing menopause experiences of autistic and non-autistic people, both shared and unique experiences were described. Shared experiences include increased psychological and somatic (bodily) symptoms, as well as difficulties disentangling menopause symptoms against a background of multiple other changes and co-occurring life events.^5^ However autistic people also described unique experiences related to challenges identifying bodily signals (interoception) and difficulties due to unpredictability of symptoms.^5^ Also notable was that lifelong experiences including interactions with healthcare professionals impacted autistic people’s experience of menopause.^5^

The increase in qualitative studies about autistic experiences of menopause has not been matched by quantitative studies or studies comparing autistic and non-autistic people’s experiences. In a recent study, autistic people (n=508) rated menopause symptoms as severe, but were rated as more severe for people who were unaware of their autism when entering menopause (compared to people who knew they were autistic,^11^). Given the known low rates of autism diagnoses among middle-aged and older autistic people, this result may suggest risk of particularly poor outcomes for undiagnosed autistic people entering menopause.^12^ To our knowledge, to date only two studies have explored whether differences exist between autistic and non-autistic people on self-reported menopause symptom questionnaires. In a study by Charlton et al.^13^ examining menopause symptoms by self-reported menopause stage for autistic (n=242) and non-autistic (n=100) people, autistic people reported higher rates of symptom severity for psychological and somatic (but not vasomotor) symptoms. It is worth noting that although depression and anxiety symptoms were not examined, including these measures as covariates in the analysis did not change the pattern of results. Another recent study by Groenman et al.^14^ explored menopause experiences among people aged over 40 years old. Autistic people (n=30) reported higher levels of psychological and somatic symptoms compared to non-autistic people (n=35), with no group differences found in urogenital symptoms.^14^ This study also demonstrated significant correlations between depressive symptoms and concurrent psychological menopause symptoms (notably, no significant correlations were identified between depression symptoms and somatic or urogenital menopause symptoms).

Findings showing associations between depression and menopause symptoms are consistent with research in the general population. Previous research suggests that preexisting health conditions, and in particular mental health conditions, increase the risk of negative experiences during menopause.^15–18^ In general population samples, lifetime history of depression and anxiety have been found to increase the risk of major depression disorder during menopause.^15,16^ Lifetime history of mood disorders may be an important risk factor for difficulties during menopause among groups where prevalence of these conditions is high. It is well known that cooccurring conditions such as anxiety and depression are common among autistic people, which may act as a risk factor increasing the likelihood of negative experiences during menopause.^19,20^ We are not aware of any study to date that has examined the relationship between pre-existing mental health conditions (or symptoms of poor mental health) and the experience of menopause symptoms for autistic people.^21^ To address this gap in the literature, the current study uses longitudinal data from the AgeWellAutism (AWA) study, collected at two timepoints approximately 4.5 years apart. We explore whether baseline self-report of depression and anxiety symptoms predicts menopause symptoms at follow-up four years later. We hypothesise that baseline mental health symptoms will predict experiences of menopause at follow-up; and that being autistic will contribute to explaining additional variance in menopause symptoms.

## Methods

### Study design

The study design is classified as conforming to STROBE criteria.^22^ This pre-registered study (https://osf.io/qc8mh/overview) uses longitudinal data from the first and second wave of the AgeWellAutism study, conducted in April 2019 and September 2023, respectively. Mean follow-up time between the first and second wave of data collection was 1599.01 days (SD = 8.69), minimum = 1585, maximum = 1614, a length of approximately 4 years and 4 months. The AgeWellAutism study is an online survey exploring ageing on the autism spectrum and was shaped by a series of patient and public involvement (PPI) interviews with 12 middle-aged and older autistic adults. Additional measures, including the menopause scale used in this study, were added in the second wave following community feedback.

Recruitment was conducted via social media platforms, research databases, Autistica’s Research Network, Cambridge Autism Research Database (CARD), and adverts in community centres and older adult residential communities. Participants who took part in the first wave consented for recontact and were invited to take part in the second wave via email. Study adverts referred to ageing and mental health, but not menopause.

Participants accessed the survey via Qualtrics and provided informed consent by endorsing items acknowledging informed consent, awareness of confidentiality and ability to withdraw from the study at any time. In both waves, participants were entered into a draw for one of twenty £20 Amazon UK gift vouchers. Inclusion criteria were being 40 years of age or above, having access to an internet-enabled device, and being able to read and type in English. The study had no specific exclusion criteria. Full ethical approval was granted by the PNM Research Ethics Subcommittee at King’s College London (HR-18/19–10941) and the University College London Research Ethics Committee (UCL-REC-25855/001) for the first and second waves, respectively.

### Participants

A combined total of 1240 responses were recorded in the AgeWellAutism study. After both the first and second waves, 179 responses were excluded in a rigorous data cleaning process involving checking for suspected spam activity, short completion times, and unusual open-text responses. This resulted in a combined total of 1061 valid responses. Of this, 322 responses (30%) were linked by study ID number, indicating that 166 participants completed both the first (W1) and second (W2) wave. Of this 166, 86 participants were further excluded from the current study due to being assigned male at birth. This resulted in a final study total of 80 participants.

The Ritvo Autism Asperger Diagnostic Scale-Revised was administered to explore consistency of self-report and autism diagnosis (RAADS,^23^; see Materials section), although note that the group assignment was based on whether individuals reported they were autistic or not, rather than the RAADS scores. The autistic group comprised of 52 participants (65%), all of whom reported having an autism diagnosis with a RAADS-14 score ≥18 (i.e., above the suggested cut-off of ≥14). All autistic participants were diagnosed in adulthood (mean years since diagnosis = 13.98, age range at diagnosis 20-77 years old). The remaining 28 participants (35%) formed a non-autistic comparison group (all but one with RAADS-14 scores ≤14). To ensure that results were not impacted by the individual without an autism diagnosis but with a high RAADS score, analyses were repeated excluding this participant.

Some statistically significant group differences were found in the demographic characteristics of the autistic and non-autistic groups. No group differences were observed in age of participants, their ethnicity, or educational history. The autistic group were more likely to be non-heterosexual, single, renting their home, and unemployed. See Table 1 for demographic characteristics of the autistic and non-autistic groups.

**Table 1.** Demographic characteristics of the autistic and non-autistic groups. Demographics about here.

​	​	Autistic group (n=52)	Non-autistic group (n=28)	Group difference	Effect size
Age (years)	M (SD)	66.48 (10.18)	63.21 (10.53)	*t*(78) = 1.35, *p* = .180	*d* = 0.32 [-0.15, 0.78]
	[95% CI]	[63.65, 69.31]	[59.13, 67.30]		
	Range	47-82	47-86		
Gender	Female	52 (100%)	28 (100%)	-	-
Sexuality	Heterosexual	35 (67.3%)	26 (92.9%)	χ2 = 6.56, *p* = .010*	V = 0.29
	Other	17 (32.7%)	2 (7.1%)		
Ethnicity	White	48 (92.3%)	23 (82.1%)	χ2 = 1.88, *p* = .170	V = 0.15
	Non-White	4 (7.7%)	5 (17.9%)		
Education history	No formal qualifications	7 (13.5%)	1 (3.6%)	χ2 = 3.04, *p* = .552	V = 0.20
	Some secondary/high school education	9 (17.3%)	4 (14.3%)		
	Secondary/high school leavers qualifications	16 (30.8%)	8 (28.6%)		
	Vocational or professional qualifications	12 (23.1%)	10 (35.7%)		
	Foundation or access-level certificate or diploma	8 (15.4%)	5 (17.9%)		
Current employment status	Employed	16 (30.8%)	19 (67.9%)	χ2 = 11.63, *p* = .009**	V = 0.38
	Student/volunteer/carer	6 (11.5%)	0		
	Unable to work	1 (1.9%)	0		
	Retired	29 (55.8%)	9 (32.1%)		
Relationship status	Married/in a civil partnership	16 (30.8%)	12 (42.9%)	χ2 = 13.38, p = .010*	V = 0.41
	In a relationship	2 (3.8%)	6 (21.4%)		
	Single	28 (53.8%)	7 (25.0%)		
	Widowed	2 (3.8%)	3 (10.7%)		
	Separated/ divorced	4 (7.7%)	0		
Living situation	Spouse or partner	23 (44.2%)	13 (46.4%)	χ2 = .04, *p* = .851	V = 0.02
	Children	20 (38.5%)	9 (32.1%)	χ2 = .31, *p* = .575	V = 0.06
	Sibling	3 (5.8%)	0	χ2 = 1.68, *p* = .195	V = 0.15
	Parent	2 (3.8%)	1 (3.6%)	χ2 = .004, *p* = .951	V = 0.007
	Other family member	2 (3.8%)	0	χ2 = 1.11, *p* = .293	V = 0.12
	Friends	4 (7.7%)	0	χ2 = 2.27, *p* = .132	V = 0.17
	Supported housing	4 (7.7%)	0	χ2 = 2.27, *p* = .132	V = 0.17
	Retirement home	13 (25.0%)	2 (7.1%)	χ2 = 3.81, *p* = .051	V = 0.22
	Alone	6 (11.5%)	11 (39.3%)	χ2 = 8.37, *p* = .004**	V = 0.32
Home Ownership	Home owned by themselves/their family	26 (50%)	26 (92.9%)	χ2 = 14.79, p = .001**	V = 0.43
	Home privately rented	8 (15.4%)	1 (3.6%)		
	Home rented from a local council/government organisation/housing association/retirement home	18 (34.6%)	1 (3.6%)		
Autism Diagnosis Age	M (SD)	52.5 (13.44)	-	-	-
	Range	20-77			
Years Since Autism Diagnosis	M (SD)	13.98 (6.16)	-	-	-
	Range	5-29			
Other Neurodiversity Diagnoses	ADHD or ADD	11 (21.2%)	0	χ2 = 6.87, *p* = .009**	V = 0.29
Menopause stage	Pre-menopausal	1 (1.9%)	5 (17.9%)	​	​
	Menopausal	10 (19.2%)	3 (10.1%)	​	​
	Post-menopausal	41 (78.8%)	20 (71.4%)	χ2 = 7.11, *p* = .029*	V = 0.30
Mean (*SD*) scores	Total Greene Climacteric Scale score	42.08 (3.70)	14.32 (3.02)	t(78) = 34.04, p < .001	*d* = 7.98 [6.64, 9.31]
	Psychological symptoms	21.96 (2.79)	6.43 (2.01)	t(78) = 25.10, p < .001	*d* = 6.09 [5.03, 7.15]
	Somatic symptoms	13.63 (2.04)	3.61 (1.29)	t(78) = 23.58, p < .001	*d* = 5.53 [4.54, 6.50]
	Vasomotor symptoms	4.38 (1.05)	2.71 (1.21)	t(78) = 6.42, p < .001	*d* = 1.51 [0.99, 2.02]
	Baseline depression	10.65 (4.85)	3.00 (2.61)	t(78) = 7.75, p < .001	*d* = 1.82 [1.27, 2.35]
	Baseline anxiety	7.54 (3.36)	2.39 (1.75)	t(78) = 7.55, p < .001	*d* = 1.77 [1.23, 2.30]
	Current depression	11.48 (5.23)	4.89 (2.75)	t(78) = 6.20, p < .001	*d* = 1.45 [0.94, 1.96]
	Current anxiety	7.96 (3.39)	3.32 (2.11)	t(78) = 6.58, p < .001	*d* = 1.54 [1.02, 2.06]
	Total RAADS-14 score	33.53 (5.97)	4.89 (4.21)	t(78) = 22.49, p < .001	d = 5.48 [4.32, 6.21]
	Mentalising score	16.63 (3.67)	2.32 (1.54)	t(78) = 19.66, p < .001	d = 3.10 [3.74, 5.46]
	Social anxiety score	9.44 (1.97)	1.89 (2.78)	t(78) = 14.09, p < .001	d = 2.28 [2.60, 3.99]
	Sensory reactivity score	7.46 (1.48)	0.76 (1.09)	t(78) = 21.22, p < .001	d = 1.36 [4.06, 5.87]

*Note*. *p < .05, **p < .01, ***p <= .001.

V=Cramer’s v; *d*=Cohen’s d.

### Materials

**Demographic information (W1 and 2).** Participants provided detailed demographic information including age, gender, ethnicity, sexuality, country of residence, education level, employment status, relationship status, and living situation. At W2, participants reported whether they considered themselves to be pre-menopausal (autistic, n=1; non-autistic, n=5), menopausal (autistic, n=10; non-autistic, n=3) or post-menopausal (autistic, n=41; non-autistic, n=20). We did not provide a definition of these stages, although menopause is considered to be the final menstrual period.^24^ As age is associated with menopause stage we include age by menopause stage and group information for reference. Autistic: pre-menopausal (age = 47, note n=1), menopausal (mean age = 52.00, SD=2.58, range = 47-54 years), post-menopausal (mean age = 70.49, SD=7.22, range = 57-82 years). Non-autistic: pre-menopausal (mean age = 48.80, SD=1.78, range = 47-51 years), menopausal (mean age = 54.00, SD=1.00, range = 53-55 years), post-menopausal (mean age = 68.20, SD=7.91, range = 57-86 years). Although menopause symptoms are most common during pre-menopause and early post-menopause, symptoms frequently last seven years.

**Current symptoms of menopause (W2 only)**. Participants reported their experience of symptoms that are associated with menopause using the Greene Climacteric Scale (GCS,^25^). The GCS is a 21-item questionnaire that asks participants to rate how bothered they are by a range of different symptoms on a 4-point scale [0 = Not at all, 1 = a little, 2 = quite a bit, 3 = extremely]. The GCS has three subscales, which examine psychological (11 items; max score = 33; e.g. “Do you feel tense or nervous?”), somatic (7 items; max score = 21; e.g. “Do you feel pains or aches in your muscles and joints?”), and vasomotor (2 items; max score = 6; e. g “Are you experiencing hot flushes?”) experiences associated with menopause. The final GCS item (interest in sex) is not included in any subscale. To the authors’ knowledge, the psychometric properties of the GCS have yet to be examined in autistic populations. In the current sample, the internal consistency of the GCS was excellent in the autistic group (Cronbach’s α = .85) and very good in the non-autistic group (Cronbach’s α = .74).

**Depression and anxiety (W1 and 2).** Symptoms of depression and anxiety were assessed using the nine-item Patient Health Questionnaire (PHQ-9,^26^) and the seven-item Generalised Anxiety Disorder questionnaire (GAD-7,^27^), respectively. Both measures use 4-point response scales to assess how often individuals have been bothered by symptoms over the past two weeks (0 = ‘not at all’ to 3 = ‘nearly all of the time’). Scores are summed to produce total scores (PHQ-9 range 0–27; GAD-7 range 0–21), with higher scores indicating greater symptom severity. Using conventional cut-off scores of ≥10, the PHQ-9 shows 88% sensitivity and 88% specificity for major depressive disorder, and the GAD-7 shows 89% sensitivity and 82% specificity for generalised anxiety disorder. Both measures have demonstrated good psychometric validity in non-autistic older adults^28,29^ and in autistic populations.^30,31^ In the current sample, internal consistency was good for both measures in the autistic group (PHQ-9 W1 α = .87, W2 α = .80; GAD-7 W1 α = .76, W2 α = .83) and good in the non-autistic group (PHQ-9 W1 α = .76, W2 α = .88; GAD-7 W1 α = .86, W2 α = .84).

**Autistic traits (W2).** Autistic traits were measured using the 14-item Ritvo Autism and Asperger Diagnostic Scale (RAADS-14; 23). Questions are scored on a 4-point scale, (3=“True now and when I was young”, 2=“True only now”,1=“True only when I was younger than 16”, 0=“Never true”). Scores are summed, and can be viewed as a total score (scores 0-42), or as three subdomains; mentalizing difficulties (scores 0-21), social anxiety (scores 0-12), and sensory reactivity (scores 0-9). Higher scores indicate a greater presence of autistic traits and characteristics. Scores ≥14 have 97% sensitivity and 95% specificity for identifying autism compared to healthy non-autistic comparisons. Autistic people without intellectual disability often score ≥32. In the current sample, internal consistency was very good in the autistic group (Cronbach’s α = .81) and acceptable in the non-autistic group (Cronbach’s α = .70).

### Statistical analysis

All statistical analyses were conducted using SPSS (version 29.0; IBM Corp, 2022). Our analysis plan was pre-registered (https://osf.io/qc8mh/overview, under embargo). After pre-registering and reviewing available measures, a decision was made to 1) omit physical health conditions due to the scale including lifetime diagnoses without information about illness severity, and to also 2) account for symptoms of depression and anxiety at follow-up in the regression models to account for current mood.

Group differences (autistic vs. non-autistic) in demographic variables were examined using t-tests (continuous) and chi-square (χ^2^) tests (categorical); adjusted residuals were used to interpret categorical results.

Linear regression models were used to examine whether autism group status predicted menopause symptoms (at follow-up) over-and-above the symptoms of poor mental health (measured at baseline). Regression models were run for each subscale of the Greene Climacteric Scale measure, first adding age (step 1), then baseline depression and anxiety symptoms (step 2), and finally, autism diagnosis status (step 3). Analyses were repeated with subscales of the RADDS entered at step 3 in place of autism diagnosis, to explore which aspects of autism may impact menopause experiences. Additional (post-hoc) regression analyses included steps 1 and 2 as above, with baseline and follow-up depression and anxiety symptoms entered at step 3, and autism diagnosis entered at step 4.

An a priori power calculation was conducted (with an alpha level of .050), indicating that at least 75 total participants per regression were required to identify medium effect sizes while maintaining 95% statistical power. Multiple comparisons were corrected using the False Discovery Rate (FDR) method,^32^ with an initial α = .05. All significant analyses survived the adjusted FDR α-threshold.

## Results

### Descriptive statistics of menopause symptoms and mental health variables

There was a significant difference in menopause stage x autism grouping (*X*^2^=7.11, *p=*0.029), with more non-autistic people than autistic people in the pre-menopausal group. No other menopause stage by autism group differences were observed. The autistic group reported significantly higher overall current menopause symptoms, as well as higher subdomain (psychological, somatic, vasomotor) symptom scores, than the non-autistic group. Additionally, the autistic group reported significantly higher self-reported symptoms of baseline and current depression and anxiety than the non-autistic group. See Table 1.

Prior to regression analyses, correlation matrices were calculated for the autistic and non-autistic participants separately. For the autistic group, significant correlations were observed between overall menopause symptoms and psychological menopause symptoms with baseline and current anxiety and depression. These associations were not significant for the non-autistic group. See Table 2.

**Table 2.** Correlations between study variables, split by autistic group (above line) and non-autistic group (below line). Correlations about here.

*Autistic above, non-autisticbelow*	1. Current age		2. Current depression		3. Current anxiety		4. Baseline depression		5. Baseline anxiety		6. Current overall menopause symptoms		7. Current psychological menopause symptoms		8. Current somatic menopause symptoms		9. Current vasomotor menopause symptoms	
1	​		r = .606	p < .001***	r = .172	p = .222	r = .358	p = .009**	r = .087	p = .541	r = -.047	p = .742	r = -.033	p = .816	r = .099	p = .484	r = -.183	p = .195
2	r = -.152	p = .439	​		r = .517	p < .001***	r = .578	p < .001***	r = .356	p = .010**	r = .329	p = .019*	r = .507	p < .001***	r = .064	p = .752	r = -.094	p = .371
3	r = -.082	p = .680	r = .555	p = .002**	​		r = .448	p < .001***	r = .666	p < .001***	r = .170	p = .228	r = .323	p = .013*	r = -.125	p = .511	r = -.163	p = .208
4	r = -.224	p = .253	r = .593	p < .001***	r = .565	p = .002**	​		r = .591	p < .001***	r = .359	p = .009**	r = .527	p < .001***	r = .054	p = .702	r = -.193	p = .171
5	r = -.127	p = .518	r = .539	p = .003**	r = .657	p < .001***	r = .827	p < .001***	​		r = .170	p = .228	r = .363	p = .008**	r = -.102	p = .471	r = -.143	p = .312
6	r = -.603	p < .001***	r = -.045	p = .821	r = .065	p = .744	r = .150	p = .445	r = .094	p = .633	​		r = .744	p < .001***	r = .573	p < .001***	r = .285	p = .041*
7	r = -.677	p < .001***	r = -.199	p = .310	r = .010	p = .960	r = .007	p = .972	r = .003	p = .988	r = .728	p < .001***	​		r = -.016	p = .909	r = -.015	p = .916
8	r = .001	p = .996	r = .092	p = .641	r = .267	p = .170	r = .331	p = .085	r = .334	p = .082	r = .358	p = .061	r = .010	p = .959	​		r = .049	p = .732
9	r = -.410	p = .030*	r = .090	p = .648	r = -.006	p = .975	r = .094	p = .636	r = .055	p = .782	r = .724	p < .001***	r = .265	p = .173	r = .068	p = .732	​	

*Note*. Cells above the diagonal indicate a significant correlation (p<.05) for the autistic group, and cells below the diagonal indicate a significant correlation (p<.05) for the non-autistic group. *p < .05, **p < .01, ***p <= .001.

### Regression models

#### Overall menopause symptoms

Step 1: Current age did not significantly contribute to the model explaining overall menopause symptoms (R^2^=.009, F=0.67, *p*=.414).
Step 2: Current age was not a significant predictor. Baseline mental health significantly contributed to the model, with depression (β=0.50, *p*<.001) and anxiety (β=0.27, *p*=.037) both contributing significantly (R^2^=.524, R^2^_change_=.516, F=27.92, *p*<.001).
Step 3 (Autism diagnosis): Baseline anxiety did not contribute significantly to the model. Current age (β=-0.08, *p*=.005), baseline depression (β=0.15, *p*=.001), and autism diagnosis (β=-0.91, *p*<.001) were significant predictors (R^2^=.949, R^2^_change_=.425, F=349.99, *p*<.001). See Table 3 for details.

Alternative Step 3 (Autistic traits): Baseline anxiety and social anxiety did not significantly contribute to the model. Current age (β=-0.14, *p*=.012), baseline depression (β=0.20, *p*=.025), mentalising difficulties (β=0.33, *p*=.022),and sensory reactivity (β=0.44, *p*=.003), were significant predictors (R^2^=.819, R^2^_change_=.295, F=55.16, *p*<.001). See Table 3 for details.

**Table 3.** Results of regression analyses exploring the impact of baseline mental health, autism diagnosis, and autistic traits on current menopause symptoms.

​	Total greene climacteric scale score		Greene climacteric scale menopause symptom subscales					
			Psychological		Somatic		Vasomotor	
Step 1	F=0.67, p=.414, R^2^=.009		F=0.45, p=.503, R^2^=.006		F=2.23, p=.139, R^2^=.028		F=1.28, p=.262, R^2^=.016	
Age	β=0.093, p=.414		β=0.076, p=.503		β=0.167, p=.139		β=-0.127, p=.262	
Step 2	F=27.92, p<.001, R^2^ =.524,		F=34.63, p<.001, R^2^=.577,		F=19.67, p<.001, R^2^=.437,		F=4.75, p=.004, R^2^=.158,	
	R^2^ change=.516		R^2^ change=.572		R^2^ change=.409		R^2^ change=.142	
Age	β=-0.074, p =.376		β=-0.100, p=.205		β=0.020, p=.829		β=-0.212, p=.058	
Baseline Depression	β=0.503, p<.001***		β=0.535, p<.001***		β=0.443, p=.003**		β=0.249, p=.160	
Baseline Anxiety	β=0.273, p=.037*		β=0.283, p=.022*		β=0.249, p=.079		β=0.159, p=.356	
Step 3 (Autism Diagnosis)	F=349.99, p<.001, R^2^=.949,	-	F=236.21, p<.001, R^2^=.926,	-	F=137.74, p<.001, R^2^=.880,	-	F=12.39, p<.001, R^2^=.398,	-
	R^2^ change=.425	-	R^2^ change=.349	-	R^2^ change=.443	-	R^2^ change=.240	-
Age	β=-0.079, p=.005**	**-**	β=-0.105, p=.002**	**-**	β=0.014, p=.745	**-**	β=-0.216, p=.024*	**-**
Baseline Depression	β=0.153, p=.001***	**-**	β=0.217, p<.001***	**-**	β=0.085, p=.227	**-**	β=-0.014, p=.927	**-**
Baseline Anxiety	β=-0.039, p=.378	-	β=-0.001, p=.991	-	β=-0.070, p=.301	-	β=-0.076, p=.617	-
Autism Diagnosis	β=-0.905, p<.001***	**-**	β=-0.820, p<.001***	**-**	β=-0.924, p<.001***	**-**	β=-0.680, p<.001***	**-**
Alternate Step 3 (Autistic Traits)	-	F=55.16, p<.001, R^2^=.819,	-	F=51.82, p<.001; R^2^=.810,	-	F=38.68, p<.001, R^2^=.761,	-	F=6.87, p<.001, R^2^=.361,
	-	R^2^ change=.295	-	R^2^ change=.232	-	R^2^ change=.324	-	R^2^ change=.203
Age	**-**	β=-0.135, p=.012*	**-**	β=-0.157, p=.005**	**-**	β=-0.043, p=.479	**-**	β=-0.255, p=.012*
Baseline Depression	**-**	β=0.204, p=.025*	**-**	β=0.281, p=.003**	**-**	β=0.125, p=.225	**-**	β=-0.024, p=.885
Baseline Anxiety	-	β=-0.040, p=.649	-	β=0.013, p=.888	-	β=-0.090, p=.373	-	β=-0.100, p=.543
Mentalising Difficulties	**-**	β=0.329, p=.022*	**-**	β=0.316, p=.032*	**-**	β=0.375, p=.023*	**-**	β=0.121, p=.649
Social Anxiety	-	β=0.058, p=.599	-	β=-0.046, p=.681	-	β=0.146, p=.250	-	β=0.256, p=.218
Sensory Reactivity	**-**	β=0.438, p=.003**	**-**	β=0.440, p=.004**	**-**	β=0.360, p=.034*	**-**	β=0.332, p=.228

*Note*. *p < .05, **p < .01, ***p <= .001.

#### Psychological menopause symptoms

Step 1: Current age did not significantly contribute to the model explaining psychological menopause symptoms (R^2^=.006, F=0.45, p=.503).
Step 2: Current age was not a significant predictor. Baseline mental health significantly contributed to the model, with depression (β=0.54, p<.001) and anxiety (β=0.28, *p*=.022) both contributing significantly (R^2^=.577, R^2^_change_=.572, F=34.63, *p*<.001).
Step 3 (Autism diagnosis): Baseline anxiety did not contribute significantly to the model. Current age (β=-0.11, *p*=.002), baseline depression (β=0.22, *p*<.001), and autism diagnosis (β=-0.82, *p*<.001) were significant predictors (R^2^=.926, R^2^_change_=.349, F=236.21, *p*<.001).

Alternative Step 3 (Autistic traits): Baseline anxiety and social anxiety did not significantly contribute to the model. Current age (β=-0.16, *p*=.005), baseline depression (β=0.28, *p*=.003), mentalising difficulties (β=0.32, *p*=.032), and sensory reactivity (β=0.44, *p*=.004), were significant predictors (R^2^=.810, R^2^_change_=.232, F=51.82, *p*<.001).

#### Somatic menopause symptoms

Step 1: Current age did not significantly contribute to the model explaining somatic menopause symptoms (R^2^=.028, F=2.23, *p*=.139).
Step 2: Current age and baseline anxiety were not significant predictors. Only baseline depression (β=0.44, *p*=.003) significantly contributed to the model (R^2^=.437, R^2^_change_=.409, F=19.67, *p*<.001).
Step 3 (Autism diagnosis): Current age and baseline depression and anxiety did not significantly contribute to the model. Only autism diagnosis (β=-0.92, *p*<.001) was a significant predictor (R^2^=.880, R^2^_change_=.443, F=137.74, *p*<.001).

Alternative Step 3 (Autistic traits): Current age, baseline depression and anxiety, and social anxiety did not significantly contribute to the model. Mentalising difficulties (β=0.38, *p*=.023), and sensory reactivity (β=0.36, *p*=.034) were significant predictors (R^2^=.761, R^2^_change_=.324, F=38.68, *p*<.001).

#### Vasomotor menopause symptoms

Step 1: Current age did not significantly contribute to the model explaining vasomotor menopause symptoms (R^2^=.016, F=1.28, *p*=.262).
Step 2: Current age and baseline depression and anxiety did not significantly contribute to the model, although the overall model was significant (R^2^=.158, R^2^_change_=.142, F=4.75, *p*=.004).
Step 3 (Autism diagnosis): Baseline depression and anxiety did not significantly contribute to the model. Current age (β=-0.22, *p*=.024), and autism diagnosis (β=-0.68, *p*<.001) were significant predictors (R^2^=.398, R^2^_change_=.240, F=12.39, *p*<.001).

Alternative Step 3 (Autistic traits): Baseline depression and anxiety, mentalising difficulties, social anxiety, and sensory reactivity did not significantly contribute to the model. Only current age (β=-0.26, *p*=.012) was a significant predictor (R^2^=.361, R^2^_change_=.203, F=6.87, *p*<.001).

All regression analyses were repeated excluding the participant in the non-autistic group with a high RAADS score. The pattern of results remained unchanged and are not reported here.

### Post-hoc regression models

#### Overall menopause symptoms

Step 1: Current age did not significantly contribute to the model explaining overall menopause symptoms (R^2^=.009, F=0.67, p=.414). See Table 4 for details.
Step 2: Current age was not a significant predictor. Baseline mental health significantly contributed to the model, with depression (β=0.50, p<.001) and anxiety (β=0.27, p=.037) both contributing significantly (R^2^=.524, R^2^_change_=.516, F=27.92, p<.001).
Step 3: Current age, baseline anxiety, and current depression and anxiety did not significantly contribute to the model. Only baseline depression (β=0.45, p=.004) was a significant predictor (R^2^=.527, R^2^_change_=.003, F=16.50, p<.001).
Step 4: Baseline anxiety and current depression did not contribute significantly to the model. Current age (β=-0.06, p=.033), baseline depression (β=0.19, p<.001), current anxiety (β=-0.12, p=.011), and autism diagnosis (β=-0.93, p<.001) were significant predictors (R^2^=.957, R^2^_change_=.429, F=268.03, p<.001). See Table 4 for details.

Alternative step 4: Current age, baseline anxiety, and social anxiety did not contribute significantly to the model. Baseline depression (β=0.32, p<.001), current depression (β=-0.28, p=.001), current anxiety (β=-0.20, p=.015), mentalising difficulties (β=0.41, p=.002), and sensory reactivity (β=0.43, p=.001) were significant predictors (R^2^=.868, R^2^_change_=.340, F=58.16, p<.001). See Table 4 for details.

**Table 4.** Results of regression analyses exploring the impact of baseline mental health, current mental health, autism diagnosis, and autistic traits, on current menopause symptoms.

​	Total greene climacteric scale score		Menopause symptom subscales					
			Psychological		Somatic		Vasomotor	
Step 1	F=0.67, p=.414, R^2^=.009		F=0.45, p=.503, R^2^=.006		F=2.23, p=.139, R^2^=.028		F=1.28, p=.262, R^2^=.016	
Age	β=0.093, p=.414		β=0.076, p=.503		β=0.167, p=.139		β=-0.127, p=.262	
Step 2	F=27.92, p<.001, R^2^ =.524,		F=34.63, p<.001, R^2^=.577,		F=19.67, p<.001, R^2^=.437,		F=4.75, p=.004, R^2^=.158,	
	R^2^ change=.516		R^2^ change=.572		R^2^ change=.409		R^2^ change=.142	
Age	β=-0.074, p =.376		β=-0.100, p=.205		β=0.020, p=.829		β=-0.212, p=.058	
Baseline Depression	β=0.503, p<.001***		β=0.535, p<.001***		β=0.443, p=.003**		β=0.249, p=.160	
Baseline Anxiety	β=0.273, p=.037*		β=0.283, p=.022*		β=0.249, p=.079		β=0.159, p=.356	
Step 3	F=16.50, p<.001, R^2^=.527,		F=20.42, p<.001, R^2^=.580,		F=11.94, p<.001, R^2^=.447,		F=3.52, p=.007, R^2^=.192,	
	R^2^ change=.003		R^2^ change=.002		R^2^ change=.009		R^2^ change=.034	
Age	β=-0.097, p=.288		β=-0.103, p=.228		β=-0.017, p=.861		β=-0.279, p=.020*	
Baseline Depression	β=0.454, p=.004**		β=0.526, p<.001***		β=0.365, p=.030*		β=0.106, p=.595	
Baseline Anxiety	β=0.274, p=.093		β=0.339, p=.028*		β=0.197, p=.262		β=0.045, p=.830	
Current Depression	β=0.092, p=.513		β=0.033, p=.803		β=0.130, p=.394		β=0.231, p=.209	
Current Anxiety	β=-0.019, p=.896		β=-0.088, p=.523		β=0.049, p=.757		β=0.117, p=.539	
Step 4 (Autism Diagnosis)	F=268.03, p<.001, R^2^=.957,	-	F=214.83, p<.001, R^2^=.946,	-	F=90.31, p<.001, R^2^=.881,	-	F=8.35, p<.001, R^2^=.407,	-
	R^2^ change=.429	-	R^2^ change=.367	-	R^2^ change=.435	-	R^2^ change=.215	-
Age	β=-0.060, p=.033*	**-**	β=-0.069, p=.026*	**-**	β=0.020, p=.665	-	β=-0.253, p=.015*	**-**
Baseline Depression	β=0.186, p<.001***	**-**	β=0.279, p<.001***	**-**	β=0.095, p=.228	-	β=-0.083, p=.636	-
Baseline Anxiety	β=0.041, p=.412	-	β=0.124, p=.028*	**-**	β=-0.038, p=.650	-	β=-0.119, p=.519	-
Current Depression	β=-0.051, p=.243	-	β=-0.099, p=.043*	**-**	β=-0.014, p=.849	-	β=0.131, p=.414	-
Current Anxiety	β=-0.116, p=.011*	**-**	β=-0.178, p=.001***	**-**	β=-0.048, p=.513	-	β=0.049, p=.767	-
Autism Diagnosis	β=-0.926, p<.001***	**-**	β=-0.855, p<.001***	**-**	β=-0.932, p<.001***	**-**	β=-0.655, p<.001***	**-**
Alternate Step 4 (Autistic Traits)	-	F=58.16, p<.001, R^2^=.868,	-	F=61.55, p<.001, R^2^=.874,	-	F=34.23, p<.001, R^2^=.794,	-	F=5.03, p<.001, R^2^=.362,
	-	R^2^ change=.340	-	R^2^ change=.294	-	R^2^ change=.348	-	R^2^ change=.169
Age	-	β=-0.060, p=.223	-	β=-0.074, p=.129	-	β=0.023, p=.704	-	β=-0.248, p=.025*
Baseline Depression	**-**	β=0.315, p<.001***	**-**	β=0.403, p<.001***	**-**	β=0.228, p=.034*	**-**	β=-0.015, p=.937
Baseline Anxiety	-	β=0.064, p=.477	-	β=0.143, p=.106	-	β=-0.022, p=.848	-	β=-0.083, p=.676
Current Depression	**-**	β=-0.280, p=.001***	**-**	β=-0.307, p<.001***	**-**	β=-0.256, p=.014*	**-**	β=-0.025, p=.890
Current Anxiety	**-**	β=-0.201, p=.015*	**-**	β=-0.249, p=.002**	-	β=-0.138, p=.175	-	β=-0.032, p=.859
Mentalising Difficulties	**-**	β=0.411, p=.002**	**-**	β=0.406, p=.001***	**-**	β=0.452, p=.005**	**-**	β=0.128, p=.641
Social Anxiety	-	β=0.173, p=.081	-	β=0.087, p=.363	-	β=0.240, p=.053	-	β=0.270, p=.214
Sensory Reactivity	**-**	β=0.429, p=.001***	**-**	β=0.431, p=.001***	**-**	β=0.347, p=.031*	**-**	β=0.332, p=.236

*Note*. *p < .05, **p < .01, ***p <= .001.

#### Psychological menopause symptoms

Step 1: Current age did not significantly contribute to the model explaining psychological menopause symptoms (R^2^=.006, F=0.45, p=.503). See Table 4 for details.
Step 2: Current age was not a significant predictor. Baseline mental health significantly contributed to the model, with depression (β=0.54, p<.001) and anxiety (β=0.28, p=.022) both contributing significantly (R^2^=.577, R^2^_change_=.572, F=34.63, p<.001).
Step 3: Current age and current depression and anxiety did not significantly contribute to the model. Baseline depression (β=0.53, p<.001) and baseline anxiety (β=0.34, p=.028) were significant predictors (R^2^=.580, R^2^_change_=.002, F=20.42, p<.001).
Step 4: Current age (B=-0.07, p=.026), baseline depression (β=0.28, p<.001), baseline anxiety (β=0.12, p=.028), current depression (β=-0.10, p=.043), current anxiety (β=-0.18, p=.001), and autism diagnosis (β=-0.86, p<.001) all contributed significantly to the model (R^2^=.946, R^2^_change_=.367, F=214.83, p<.001).

Alternative step 4: Current age, baseline anxiety, and social anxiety did not contribute significantly to the model. Baseline depression (β=0.40, p<.001), current depression (β=-0.31, p<.001), current anxiety (β=-0.25, p=.002), mentalising difficulties (β=0.41, p=.001), and sensory reactivity (β=0.43, p=.001) were significant predictors (R^2^=.874, R^2^_change_=.294, F=61.55, p<.001).

#### Somatic menopause symptoms

Step 1: Current age did not significantly contribute to the model explaining somatic menopause symptoms (R2=.028, F=2.23, p=.139). See Table 4 for details.
Step 2: Current age and baseline anxiety were not significant predictors. Only baseline depression (β=0.44, p=.003) significantly contributed to the model (R^2^=.437, R^2^_change_=.409, F=19.67, p<.001).
Step 3: Current age, baseline anxiety, and current depression and anxiety did not significantly contribute to the model. Only baseline depression (β=0.37, p=.030) was a significant predictor (R^2^=.447, R^2^_change_=.009, F=11.94, p<.001).
Step 4: Current age, baseline depression and anxiety, and current depression and anxiety did not significantly contribute to the model. Only autism diagnosis (β=-0.93, p<.001) was a significant predictor (R^2^=.881, R^2^_change_=.435, F=90.31, p<.001).

Alternative step 4: Current age, baseline anxiety, current anxiety, and social anxiety did not contribute significantly to the model. Baseline depression (β=0.23, p=.034), current depression (β=-0.26, p=.014), mentalising difficulties (β=0.45, p=.005), and sensory reactivity (β=0.35, p=.031) were significant predictors (R^2^=.794, R^2^_change_=.348, F=34.23, p<.001).

#### Vasomotor menopause symptoms

Step 1: Current age did not significantly contribute to the model explaining vasomotor menopause symptoms (R2=.016, F=1.28, p=.262). See Table 4 for details.
Step 2: Current age and baseline depression and anxiety did not significantly contribute to the model, although the overall model was significant (R^2^=.158, R^2^_change_=.142, F=4.75, p=.004).
Step 3: Baseline depression and anxiety, and current depression and anxiety, did not significantly contribute to the model. Only current age (β=-0.28, p=.020) was a significant predictor (R^2^=.192, R^2^_change_=.034, F=3.52, p=.007).
Step 4: Baseline depression and anxiety, and current depression and anxiety, did not significantly contribute to the model. Current age (β=-0.25, p=.015) and autism diagnosis (β=-0.66, p<.001) were significant predictors (R^2^=.407, R^2^_change_=.215, F=8.35, p<.001).

Alternative step 4: Baseline depression and anxiety, current depression and anxiety, mentalising difficulties, social anxiety, and sensory reactivity did not contribute significantly to the model. Only current age (β=-0.25, p=.025) was a significant predictor (R^2^=.362, R^2^_change_=.169, F=5.03, p<.001).

Regression analyses were repeated excluding the participant in the non-autistic group with a high RAADS score. The pattern of results remained unchanged and are not reported here.

## Discussion

This study explored whether baseline self-reported depression and anxiety symptoms predicted menopause symptoms at four-year follow-up for autistic and non-autistic people. Pre-existing depression symptoms were a risk factor for later total, psychological and somatic menopause symptoms (but not vasomotor symptoms). Anxiety symptoms contributed to explaining the variance in total and psychological symptoms but not somatic or vasomotor symptoms. These results are in keeping with results from the general population demonstrating the importance of depression and anxiety as risk factors for difficulties during menopause and vice versa.^16,18,33^ When autism diagnosis was included in the models, it was a significant predictor of severity of menopause symptoms in the total score and across all sub-scales (psychological, somatic and vasomotor). Notably, when autism diagnosis was included in the regression models, it was a stronger predictor of symptom severity than depression, although depression remained a significant predictor in the model for total and psychological symptoms. Results are in line with results from previous studies suggesting that autistic people may have challenges during menopause above those experienced by non-autistic peers.^5,11,13,14^ It is worth noting that age did not contribute significantly to the regression analyses, until Step 3 of the models when it was a significant contributor to the models for total, psychological and vasomotor symptoms. For these models, younger age contributed to higher total, psychological and vasomotor symptoms. A possible explanation for this, is that younger people experiencing menopause are likely to find menopause symptoms more challenging, and this is exacerbated by a history of depressive symptoms and being autistic.^4,20,34^ Studies in the general population demonstrate that menopause symptoms increase with age, but that early experience of menopause (for a variety of reasons) increases individual health and psychological risk.^34–36^ An alternative explanation is that the younger group were more likely to be currently experiencing perimenopause or menopause. Age alone was not a significant contributor to the regression models predicting menopausal symptoms, and was only significant when autism group was included. It is worth noting that the correlation matrices demonstrate that age correlates significantly with overall menopause symptoms and the psychological and vasomotor subscales for non-autistic people (indicating more symptoms at a younger age). However, no significant correlations between age and menopause symptoms are observed in the autism group. It is also notable that in a previous analysis (the current sample is a sub-set of a larger sample), menopause symptoms were reported at similar levels during menopause and post-menopause stages for the autistic group but not the non-autistic group.^13^ We previously hypothesised that trajectories of menopause symptoms may not be the same for autistic and non-autistic people, which may explain the lack of age associations with menopause symptoms for the autistic group. However, this finding will need to be validated in an independent sample. Importantly for the planned analysis, results support emerging evidence that history of depression and being autistic may be risk factors for negative menopause experiences with autism diagnosis explaining variance in psychological, somatic and vasomotor symptoms.

In order to explore whether specific autistic traits were associated with menopause difficulties, the regression analyses were repeated including the RAADS-14 subscales of mentalising difficulties, social anxiety and sensory reactivity.^23^ The results show a similar pattern to regressions including diagnostic status, with the autistic traits of mentalising difficulties and sensory reactivity contributing significantly to explaining the models for total, psychological and somatic symptoms. For total and psychological symptoms, younger age, higher depression symptoms and more mentalising difficulties and sensory reactivity predicted more difficulties with menopause symptoms. The autistic traits of mentalising difficulties and sensory reactivity were the only significant predictor of somatic symptoms (the same pattern of results as when including autism diagnosis). The inclusion of mentalising difficulties as an aspect of autism that significantly explained menopause symptoms may at first seem surprising. However, the mentalising difficulties subscale includes items relating to social interactions (figuring out what people expect) which may impact interactions with healthcare professionals, and being upset by sudden changes with unpredictable changes being common during menopause. Notably, difficulties interacting with healthcare professionals and challenges related to unpredictable menopause symptoms have both been described in previous qualitative studies.^4,5,11^ The subscale of sensory reactivity includes items relating to textures and sensory overwhelm, which may show particular links to somatic and psychological symptoms.^4,8,37^ For the model exploring variables impacting vasomotor symptoms, although younger age and autism diagnosis contribute significantly to the original model, when including autistic traits only younger age contributed significantly to the model. This suggests that although being autistic contributed to severity of vasomotor symptoms, the individual autistic traits measured here (mentalising difficulties, social anxiety, sensory reactivity), were not significant factors predicting vasomotor symptoms. This may reflect vasomotor symptoms being very specific in terms of sensory experience and impact, where individual subscales do not include items reflecting these experiences. Results instead suggest that younger age was an important predictor of severity of vasomotor experiences. Literature in the general population suggests that early onset of vasomotor symptoms is an important risk factors for mental and physical health.^38,39^ In general, a similar pattern of results was observed including autism diagnosis compared to autism traits in the regression models. It is worth noting that social anxiety did not contribute independently to the models, whereas mentalising difficulties and sensory reactivity did. Future studies could consider whether specific autistic traits are more associated with different menopause difficulties.

To account for the impact of current depression and anxiety, regression analyses were repeated adding depression and anxiety symptom scores at follow-up (in addition to baseline measures). Notably, current depressive and anxiety symptoms did not contribute significantly at Step 3 to any of the models (total, psychological, somatic, vasomotor) after accounting for prior history of these symptoms. Partly this result may be accounted for by the shared variance between baseline and current symptoms, but it also raises important questions about the impact of lifelong conditions during menopause transition.^15–18^ With the addition of autism diagnosis in the final step of the model, both baseline and current mood (for total score: current depression; for psychological symptoms: current depression and anxiety) were included in the model, along with age and autism diagnosis. The regression results indicate different patterns of association for baseline (positive beta weights) versus current (negative beta weight) mood, with the impact of autism diagnosis also changing direction compared to the models excluding current mood. This pattern of results may reflect the impact of a relatively small sample size and multicollinearity of variables. However, repeated measures of mood seem to be interacting with autism diagnosis in some way. It is worth noting that a previous analysis of menopause symptoms (using an overlapping dataset to that included here), suggested that symptoms were not lower in post-menopause (compared to menopause) for autistic people, in contrast to fewer symptoms in post-menopause (compared to menopause) for non-autistic people. Whether a differences in symptoms changing (or not) over time is impacting regression results, requires further exploration. A similar pattern of results is noted when including subscales of the RAADS-14 in place of autism diagnosis. Further longitudinal research with larger groups is required to better understand how historic and current mood symptoms may impact menopause experiences for autistic people.

## Limitations

Overall, results suggest a similar pattern of results among autistic people to those found in the general population, where pre-existing depression and anxiety are risk factors for a range of negative symptoms related to menopause transition, although a bidirectional relationship may also exist.^17,18,38,39^ However, limitations of this study should be considered. Data was collected as part of an online study potentially introducing bias, as online study participants must have access to a computer and internet, and may be of higher socioeconomic status or more highly educated. Therefore, study participants may not be representative of the population as a whole. As the study was designed to explore middle-age and later-life, this may reduce recruitment bias (i.e. not focusing only on those who think menopause is problematic). Information on menopause stage was limited and self-described as pre-menopausal, menopausal or post-menopausal. Most participants (76%) describe themselves post-menopausal, although this does not equate to being symptoms free.^24^ Future studies should include date of last menstrual period to better classified menopause stage. The sample size was adequate (according to a power calculation) but modest, and uneven group sizes may have contributed to difficulties interpreting interactions with diagnostic group. Despite these limitations, the advantage of the study is that it includes longitudinal data allowing exploration of variables that explain bothersome menopause symptoms.

## Conclusion

In conclusion, results align overall with research in the general population, suggesting a history of depression increases menopause related difficulties and that being autistic is an additional risk factor explaining bothersome psychological, somatic and vasomotor menopause symptoms. To our knowledge, this is the first study to explore factors influencing future menopause symptoms in autistic populations. Further large-scale research should examine longitudinal change and the impact of other variables of interest.

## Data Availability

The data that support the findings of this study are held by Dr. Gavin Stewart, but the availability of these data is restricted. The data were used under license for the current study and are not publicly available. However, the data may be available from the authors upon reasonable request and with permission from Dr. Gavin Stewart.*
